# Nanosilicon: An approach for abiotic stress mitigation and sustainable agriculture

**DOI:** 10.3389/fpls.2022.1025974

**Published:** 2022-12-20

**Authors:** Krishan K. Verma, Yuan Zeng, Xiu-Peng Song, Munna Singh, Kai-Chao Wu, Vishnu D. Rajput, Yang-Rui Li

**Affiliations:** ^1^ Key Laboratory of Sugarcane Biotechnology and Genetic Improvement (Guangxi), Ministry of Agriculture and Rural Affairs/Guangxi Key Laboratory of Sugarcane Genetic Improvement/Sugarcane Research Institute, Guangxi Academy of Agricultural Sciences, Nanning, Guangxi, China; ^2^ International Co-operation Division, Guangxi Academy of Agricultural Sciences, Nanning, Guangxi, China; ^3^ Department of Botany, University of Lucknow, Lucknow, India; ^4^ Academy of Biology and Biotechnology, Southern Federal University, Rostov-on-Don, Russia

**Keywords:** leaf gas exchange, enzymatic and non-enzymatic activities, abiotic stress, nano-silica, stress relief, environmental health

## Abstract

Abiotic stresses cause extensive yield loss in various crops globally. Over the past few decades, the application of silicon nanoparticles (nSi) has emerged as an abiotic stress mitigator. The initial responses of plants are exemplified by the biogenesis of reactive oxygen species (ROS) to sustain cellular/organellar integrity, ensuring *in vivo* operation of metabolic functions by regulating physiological and biochemical pathways during stress conditions. Plants have evolved various antioxidative systems to balance/maintain the process of homeostasis via enzymatic and non-enzymatic activities that repair any losses. In an adverse environment, supplementation of Si mitigates the stress condition and improves the growth and development of plants. Its ameliorative effects are correlated with enhanced antioxidant enzymes activities, maintaining the equilibrium between ROS generation and reduction. However, a limited number of studies cover the role of nSi in abiotic stress conditions. This review addresses the accumulation and/or uptake of nSi in several crops, as well as its mode of action, which are linked with improved plant growth and tolerance capabilities, contributing to sustainable agriculture.

## Introduction

Sustainable agriculture is a major economic sector associated with a wide range of food crops. Finding sustainable solutions for crop adaptation strategies to adverse environmental conditions and enhancing crop production are key to guaranteeing food security and safety worldwide. Abiotic stresses damage plant productivity of food crops by approximately 51–82% annually. Farmers regularly use pesticides and synthetic fertilizers to enhance crop production, which pose threats to the agricultural ecosystem. However, this approach may assist plant performance/fitness for crop improvement and increased fruit and grain quality (Verma et al., 2022a; Wang et al., 2022) during adverse environmental conditions. The enhancement of crop production is an emerging interdisciplinary area that can potentially promote plant growth and stress tolerance.

Nanoparticles (NPs) are 1–100 nm in size and have unique physiological features such as a large surface area, enhanced solubility, and translocation/uptake in an entire plant system. Several NPs, for example, Fe_3_O_4_, MgO, SiO_2_, and CeO_2_ are beneficial for plant development, playing an essential role in enhancing the seed germination rate and plant tolerance, reducing pesticide residues, and improving soil fertility (Salajegheh et al., 2020; Verma et al., 2022b; Wang et al., 2022). Silicon (Si) strongly bonds with oxygen in the earth, and its uptake was found to be approximately 0.1–10% (dry weight basis) in terrestrial plants (Epstein, 2009; Mathur and Roy, 2020). Si is available as silicates, oxides, aluminum silicates, and silica (SiO_2_), and these forms are easily accessible to plants since they are naturally available in the rhizosphere. Silicon is not considered an essential element for plant growth and development. It is classified as a ‘multi-talented,’ quasi-essential element due to its important role in physiological/metabolic pathways, cell structure, and plant survival during adverse environmental conditions. Among the various types of NPs, nSi has exhibited a significant ability to enhance plant performance in stressful conditions (Liang et al., 2015; Hussain et al., 2019; Verma et al., 2022d).

Soil fortification using nano-materials (NMs) is a trending development. Scientific groups have demonstrated various novel nano-stabilizers for soil improvement technologies. Among the various well-established additives (Kannan and Sujatha, 2022), nSi shows remarkable performance in *Hordeum vulgare*, *Phaseolus vulgaris*, *Cucumis sativus*, and *Saccharum officinarum* crops (Yassen et al., 2017; Elsheery et al., 2020; Ghorbanpour et al., 2020; Koleva et al., 2022). Studies have also suggested that nSi, as a sole-additive, boosts soil fertility. Furthermore, nSi helps in reducing the hydraulic conductivity and compression index of the soil (Kannan and Sujatha, 2022).

Recent investigations have indicated that the fertilization of soil with nSi stimulates the photosynthetic CO_2_ assimilation rate as well as biochemical and molecular responses in plants that resist unfavorable environmental conditions (Verma et al., 2022a). Abiotic stresses induce the generation of reactive oxygen species (ROS), i.e., singlet oxygen, superoxide, hydrogen peroxide, and hydroxyl radicals in cells (Das and Roychoudhury, 2014; Kim et al., 2017). ROS can cause severe oxidative injury to the protein, DNA, and lipids of the cell components. This review explores developments in crop improvement based on the existing literature and the current understanding of the action mechanisms of nSi in response to abiotic stresses, which improve physiological fitness/performance associated with sustainable agriculture.

## Advantages and disadvantages of silicon nanoparticles

**Figure 1:**
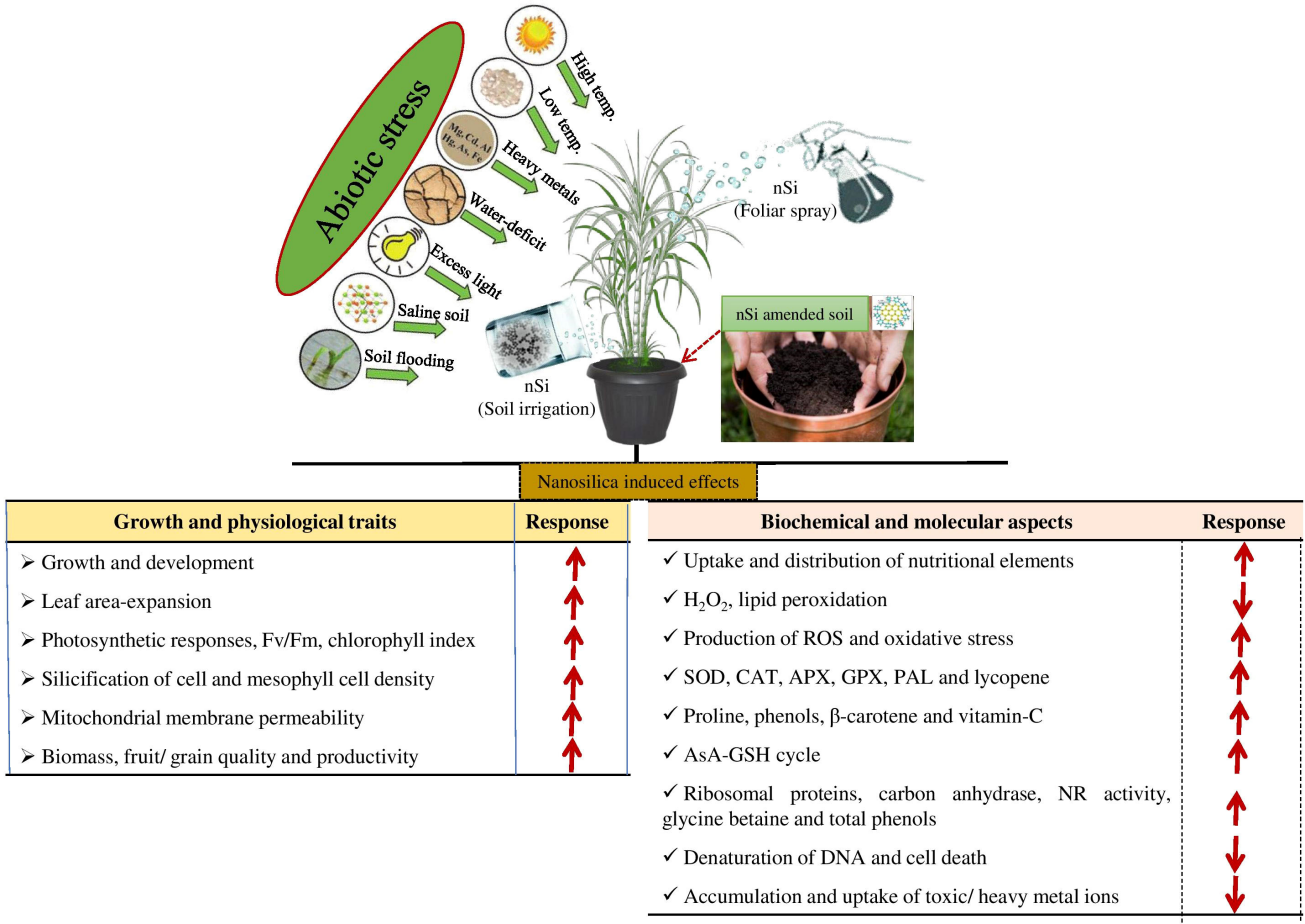
The potential mechanisms of nanosilicon (nSi) on plant physiological, biochemical, and molecular responses during unfavorable environmental conditions.

There is growing pressure on the agricultural sector to fulfill the requirements of the increasing human population. Synthetic fertilizers are indispensable in enhancing plant production and they are extensively applied through different approaches (Feregrino-Perez et al., 2018). However, plants utilize less than half of fertilizers applied and the remaining minerals may leach down, so that they become fixed in rhizospheric soil, contributing to water pollution (Liu and Lal, 2015; Zulfiqar et al., 2019). The uneven use of fertilization, without control of nutrient release, may affect crop quality. Thus, it is important to design slow/controlled-release fertilizers to sustain agricultural productivity (Rajput et al., 2021; Verma et al., 2022a). Consequently, the unique properties of NPs have attracted considerable attention in sustainable agriculture and environmental protection. Differences in the physical and chemical properties of nSi relative to their bulk counterparts occur due to their small size, higher surface area/weight ratio, and structure (Mathur and Roy, 2020). Recent findings showed better performance in plant development and alleviating environmental stresses when using nSi (Bhat et al., 2021; Verma et al., 2022b) (**Figure 1** and **Table 1**).

**Table 1:** Summary of the effects of nanosilicon (nSi) for the management of unfavorable environmental conditions in a variety of crops.

Stress	Plant	Treatment condition	Concentration range	Impacts	Source
Heavy metal (Cd)	Common bean (*Phaseolus vulgaris*)	Seed priming	20 ppm	Upregulated growth, photosynthetic leaf gas exchange efficiency, and downregulated MDA and EL levels. Enhanced K^+^ content, biosynthesis of polyamines (PAs), antioxidative enzymatic activities, and higher spermidine (Spd) and putrescine (Put) levels.	Koleva et al., 2022
Heavy metal (Cd)	Wheat (*Triticum aestivum*)	Soil amendment and foliar	300–1200 m kg^−1^	Plant biomass, photosynthetic pigments, and leaf gas exchange responses upregulated significantly, whereas oxidative stress, accumulation, and uptake of Cd considerably reduced.	Ali et al., 2019
Heavy metal (Pb and Cd)	Rice (*Oryza sativa*)	Foliar	5–20 ppm	Positively improved productivity and quality of rice grains by downregulating Cd and Pb uptake.	Hussain et al., 2020
Heavy metal (Cd)	Coriander (*Coriandrum sativum*)	Foliar	1.5 mM	Enhanced the rate of germination (%), photosynthetic efficiency, and antioxidant defense system.	Fatemi et al., 2020
Heavy metal (Pb)	Bamboo (*Pleioblastus pygmaeus*)	Nodal explants (pre-treatment)	100–500 µM	Enhanced the efficiency of SOD, CAT, GR, and PAL during stress. Can protect the plasma membrane and preserve the integrity of cells against stress by decreasing H_2_O_2_, SP, and PPO content. NPs increased plant growth/biomass/productivity by enhancing the antioxidative enzymatic activities during Pb stressed conditions.	Emamverdian et al., 2019
Heavy metal (As)	Maize (*Zea mays*)	Applied in nutrient solution (*in vitro* condition)	10 µM	Mitigated the As toxicity in maize plants, which could be associated with reduced As accumulation and oxidative stress, and increased ascorbate-glutathione cycle (AsA-GSH cycle).	Tripathi et al., 2016
Heavy metal (Hg)	Soybean (*Glycine max*)	Hydroponic	30–50 nm	Upregulated growth reduction and reduced the accumulation and translocation of Hg in soybean roots (62–84%), stems (68–76%), and leaves (45–71%). Increased chlorophyll content (15–50%) and enzymatic activities (21–33%) in response to Hg stressed conditions.	Li et al., 2020
Salinity	Common bean (*Phaseolus vulgaris*)	Petri dish	100–300 ppm	Improved percentage of germination (~20%), vigor index (~81%), germination efficiency (~23%), shoot length (11%), root length (23%), SDM (~111%), and RDM (328%).	Alsaeedi et al., 2017
Salinity	Strawberry *(Fragaria × anansa* cv. *Camarosa*)	Soil amendment	50–100 ppm	Maintained epicuticular wax structure, photosynthetic pigments, and carotenoid content and reduced proline content during stressed condition. Enhanced irregular (smoother) crystal wax deposits in the epicuticular layer.	Avestan et al., 2019
Salinity	Maize (*Zea mays*)	Seed priming	55–75 nm	nSi-primed seeds showed a higher germination capacity and seedling vigor index and the antioxidative enzymatic activities were upregulated which in turn suppressed the enhancement of ROS and reduced the MDA level. Overall, primed seeds improved the metabolic processes during high salinity.	Naguib and Abdalla, 2019
Water-deficit	Maize (*Zea mays*)	Foliar	100–200 ppm	Enhanced the nutrient absorption efficiency. No significant impact on P, Ca, Na, and Cu elements in the seeds, or on Ca and Na in the shoots.	Aqaei et al., 2020
Water-deficit	Olive (*Olea Europaea* cv. Kalamata)	Foliar	150-200 ppm	Improved productivity, fruit quality, and weight and also minimized the fruit drop percentage. Downregulation of proline, soluble sugars, and ABA levels, with less membrane injury expressed as MDA, H_2_O_2_, and EL.	Hassan et al., 2022
Water-deficit	Barley (*Hordeum vulgare*)	Soil amendment	125–250 ppm	The lower dose of nSi (125 ppm) was accompanied by a wider distribution of nSi in cells, and formation of a regular porosity pattern in roots. Total chlorophyll (up to 17.1%) and carotenoid (up to 24.1%) content significantly enhanced. Soil amendment showed promising potential for post-stress recovery of plants through changing morphological, physiological, and enzymatic activities.	Ghorbanpour et al., 2020
Ultraviolet-B	Wheat (*Triticum aestivum*)	Applied in nutrient solution (*in vitro* condition)	10 µM	Protected wheat seedlings during UV-B radiation by regulating oxidative stress through increased antioxidative activities.	Tripathi et al., 2017
Low Temp.	Sugarcane (*Saccharum officinarum*)	Foliar	5–15 nm	Maintained the maximum chlorophyll fluorescence efficiency of PSII (Fv/Fm), maximum photo-oxidizable PSI (Pm), photosynthesis efficiency, and enhanced NPQ and light harvesting pigments.	Elsheery et al., 2020
High Temp.	Wheat (*Triticum aestivum*)	Seed priming	1.66 mM	Maintained photosynthetic efficiency as regulated by enhancements in the photochemical efficiency of PSII and the performance index, as well as chlorophyll contents. Reduced MDA content significantly correlated to the membrane stability index.	Younis et al., 2020
Mineral nutrient	Safflower (*Carthamus tinctorius*)	Foliar	20 mM	Enhanced leaf area-expansion, development, photosynthetic pigments, and antioxidative enzymes.	Janmohammadi et al., 2016

ABA, abscisic acid; MDA, malondialdehyde; H_2_O_2_, hydrogen peroxide; ROS, reactive oxygen species; SOD, superoxide dismutase; CAT, catalase; GR, glutathione reductase; PAL, phenylalanine ammonia-lyase; SP, soluble protein; PPO, polyphenol oxidase; Fv/Fm, maximum chlorophyll fluorescence efficiency of PSII; NPQ, nonphotochemical quenching of PSII; EL, electrolyte leakage; Spd, spermidine; Put, putrescine; PAs, polyamines; SDM, shoot dry mass; RDM, root dry mass.

NPs may also have toxic effects on sustainable crop production, depending on various factors such as size, concentration, stability, application, and synthesis method (Rajput et al., 2021). A higher concentration of nSi reduced *Triticum aestivum* plant growth by affecting enzymatic and non-enzymatic activities, i.e., reduced photosynthetic pigments, lipid peroxidation (MDA), and enhanced antioxidative enzymatic responses (Karimi and Mohsenzadeh, 2016). Similar results were also noted in Bt-transgenic cotton (Le et al., 2014). With increasing concentration of nSi, the germination efficiency (%), root development, chromosomal aberration, and decrease in the mitotic index in *Allium cepa* were all affected (Silva and Monteiro, 2017). The application of nSi improved the root length, root volume, and dry mass of shoots and roots of *O. sativa* plants (Adhikari et al., 2013), while no phytotoxic effects were found in potato tubers (Mushinskiy et al., 2018). nSi was found to be toxic toward a number of bacterial species, viz., *Bacillus subtilis*, *Escherichia coli*, and *Pseudomonas fluorescens* (Jiang et al., 2009). Nanofertilizers can solve some limitations of biofertilizers, but this technology still requires further research and development.

## Synthesis, characteristics, and absorption of nanosilicon

Nanosilicons are one of the most advanced innovations of nanoscience, and, in many regards, are more efficient than their bulk silica counterparts (Jeelani et al., 2019). Naturally, nSi exists in numerous crystalline forms, such as sand and quartz, while SiO_4_ units are arranged in a tetrahedral geometry, with various architectural compositions. Common types of nSi may be synthesized and separated in various forms such as monodisperse spherical, hollow, porous, etched, and colloidal molecules (Jeelani et al., 2019; Mathur and Roy, 2020). The synthesis of nSi from agricultural waste is cost-effective and sustainable for NM production. Mesoporous nSi (5–30 nm) with pore sizes of 3–9 nm has been synthesized by the precipitation of rice husk (Liou and Yang, 2011; Gu et al., 2015). Bentonite clay was found to be a good source of nSi (Zulfiqar et al., 2016), which may also be synthesized from tetraethyl orthosilicate (Pham et al., 2017).

Nanoparticles can be absorbed by plant roots or leaves. The uptake and accumulation of NPs may differ from plant to plant, depending on their morphology, various uptake mechanisms, transport, and allocation in certain plant organs which may activate defense-responsive mechanisms against the NPs (Verma et al., 2022c). Si can be applied as a foliar spray input on the plant leaves, or directly to the root system as a basal dressing. Foliar spray may enter into the leaves and be transported to different plant organs through the cuticular or stomata (**Figure 1**). The transport of solutes *via* the cuticle may occur through lipophilic pathways for non-polar solutes *via* diffusion and penetration, and hydrophilic pathways for polar solutes *via* water pores (Wang et al., 2022).

## Application of nanosilicon for abiotic stress tolerance

Environmental stresses are major factors for plant productivity, have detrimental effects on plant development, and are a big problem for food security and safety worldwide (Verma et al., 2022a). This has urged botanists/agriculturists to enhance plant production by ~70% in the next three decades to overcome the present yield-limiting factors and to improve resource use efficiency. Recently, various studies have indicated that the application of nSi can positively reduce adverse responses to abiotic stresses such as soil texture, structure, clay minerals, pH, cation exchange capacity, soil organic matter, and soil microbial community, which affect the dispersion, aggregation, stability, solubility, bioavailability, and uptake or distribution of nSi (Rajput et al., 2021; Verma et al., 2022b). The application of nSi also mitigates the negative responses of abiotic stresses by upgrading the plants’ photosynthetic, antioxidative, and cellular processes. These comprehensive findings on the efficiency of nSi in mitigating adverse environmental conditions are shown in **Figure 1** and **Table 1**.

## Salt stress

The supplementation of nSi improved the photosynthetic leaf gas exchange, water-use efficiency (WUE), chlorophyll fluorescence yield of PSII, and photosynthetic pigments during saline stress conditions, thereby conferring increased stress resistance efficiency (Rajput et al., 2021; Verma et al., 2022a). During saline stress, nSi was shown to promote the germination (%) efficiency/rate, vigor index, plant biomass, root development, plant length, and leaf area expansion in cucumber plants (Alsaeedi et al., 2018). nSi enhanced the root and plant length of *Glycine max* (Farhangi-Abriz and Torabian, 2018), and the photosynthetic pigments also increased in *Cynodon dactylon* with the increasing level of salinity (Sharifiasl et al., 2019). In response to saline-sodic soils, foliar spray of nSi also enhanced the photosynthetic pigments, productivity, and grain quality in *Oryza sativa* plants (Kheir et al., 2019). It also maintains the deposition of epicuticular wax on the plant leaf surfaces and their stress tolerance capacity (Rajput et al., 2021).

The potential of nSi in seed priming subjected to saline conditions was demonstrated by the promotion of the germination rate and seedling vigor index, through upregulating the antioxidant enzyme activities, which, in turn, suppress the ROS increase and decrease the MDA content (Naguib and Abdalla, 2019). In natural conditions, plants produce ROS in cell organelles during photosynthetic and respiration processes. Plants may balance homeostasis *via* enzymatic and non-enzymatic detoxification mechanisms. nSi enhanced leaf proline and free amino acids to resist the penetration of NaCl in *O. sativa* and *Ocimum basilicum* plants (Abdel-Haliem et al., 2017; Kalteh et al., 2018). nSi reduced the influence of salt ions in plants by reducing Na^−^ absorption (Abdel-Haliem et al., 2017; Kalteh et al., 2018). However, nSi activated defense-related enzymes in plants under saline conditions to alleviate injury caused by ROS accumulation (Naguib and Abdalla, 2019). The expression of stress-related genes (*RBOH1*, *MAPK2*, *APX2*, *ERF5*, *MAPK3*, and *DDF2*) was found to be reduced with increasing stress resistance capacity in *Solanum lycopersicum* (Almutairi, 2016; Wang et al., 2022) (**Figure 1** and **Table 1**).

## Water-stress

Using nSi in soil amendment, soil irrigation, and foliar spray effectively increased plant productivity and fruit/grain quality during stress conditions and maintained root development and photosynthetic CO_2_ assimilation (Rajput et al., 2021; Verma et al., 2022a). Water stress in general causes a reduction in the uptake of minerals such as nitrogen, sodium, calcium, iron, zinc, copper, manganese, silicon, etc. However, nSi upregulates nitrogen, potassium, and other nutrient uptake in *T. aestivum* plants during water stress (Aqaei et al., 2020; Mathur and Roy, 2020), with aggregation of nSi in plant leaves, which initiated stomatal closure to prevent water loss in *Hordeum vulgare* (Ghorbanpour et al., 2020). Water stress enhances ROS production, leading to the overproduction of MDA and causing plants to suffer oxidative damage. nSi enhanced antioxidative enzyme activities and reduced MDA concentration during stress (Rajput et al., 2021; Verma et al., 2022b) (**Table 1**).

## Heavy metal stress

Excess uptake and accumulation of heavy metals (HMs) such as Pb, Cu, Cd, Cr, Hg, etc., in plant tissues *via* plant roots severely affect plant growth and development (Verma et al., 2022b). HMs persist in the soil for a long duration due to their adherent qualities; therefore, the soil is considered the major sink for HMs (Borah and Deka, 2023). Once HMs enter into the soil, they may percolate deep beneath the ground, leading to groundwater contamination. Monitoring and assessment of HM-associated risk factors in contaminated lands are essential for adopting a risk-based remediation approach (Borah et al., 2021). HMs adversely affect the soil’s biological properties and hinder ecosystem processes. Soil enzyme activities are considered one of the most reliable indicators of the biological state of soil and can also be used to measure the collective metabolism of the terrestrial ecosystem (Chakravarty and Deka, 2021; Kalita et al., 2022). However, soil enzyme activities such as phosphatase, urease, cellulase, dehydrogenase, and others are sensitive to the presence of HMs (Chakravarty and Deka, 2021; Borah and Deka, 2023).

nSi minimizes Cd accumulation in rice grains (by up to 30–60%), with enhanced translocation of potassium, magnesium, and iron (Chen et al., 2018). The interactive impact of Cd, Pb, Cu, and Zn in *O. sativa* cultivars with the use of nSi has revealed the potential of nSi to reduce the uptake of toxic ions in the grains (Wang et al., 2016). Soil amendment and foliar spray employing nSi were found to enhance agronomic characteristics and antioxidative enzymatic activities. The upregulated activities of antioxidant enzymes led to a reduction of H_2_O_2_, membrane MDA, and electrolyte leakage of primed seeds with increasing concentrations of nSi (Wang et al., 2022). The uptake of Cd in the roots and shoots of *O. sativa* plants was reduced by the interactive application of nSi and TiO_2_ NPs (Rizwan et al., 2019).

nSi increases the phytoremediation of Cd and Pb in *Secale montanum* plants (Moameri and Khalaki, 2019). The addition of nSi to the soils reduced the levels of extractable, exchangeable, and carbonate-bound Cd in the soil, thereby decreasing the metal toxicity (Mathur and Roy, 2020). The application of nSi increased the aluminum resistance in *Zea mays* by enhancing the uptake of nSi (de Sousa et al., 2019), significantly mitigated arsenic toxicity in *Zea mays* (Tripathi et al., 2016), and downregulated the expression of some HM transport genes, i.e., *OsLCT1* and *OsNramp5*, decreasing the uptake and translocation of toxic metals in *O. sativa* (Cui et al., 2017).

## Heat stress

Heat stress is the most crucial factor that affects plant growth and yield globally. Applying nSi effectively restored the heat stress-provoked ultracellular distortions in cellular organelles. nSi increased the photosynthetic efficiency, as revealed by the enhancement in the photochemical efficiency of PSII performance with chlorophyll content. Downregulation of MDA accumulation in nSi-applied plants was found to be correlated to their membrane stability index (Sun et al., 2014; Younis et al., 2020). However, prior treatment of *T. aestivum* with nSi reduced heat stress-induced negative ultrastructural variations, as revealed by the improved integrity of the nuclear envelope and the normal dispersion of chromatin (Younis et al., 2020). A high reactivity characterized the nSi binding affinity with PS II, which stabilized the photosynthetic activity during stress (Noji et al., 2010) and regulated the integrity of cell walls and membranes due to deposition of nSi at the interface of the plasma membrane-cell wall and/or in intercellular spaces (Bauer et al., 2011; Sun et al., 2014; Asgari et al., 2018) (**Figure 1** and **Table 1**).

## Cold stress

Cold stress causes significant loss to plant production in arid and semi-arid areas (Selvarajan et al., 2018). During cold stress, foliar application of nSi upregulated the chlorophyll fluorescence yield of PSII (Fv/Fm) and the maximum photo oxidizable P_700_ (Pm) activity. The improved Fv/Fm and Pm activity indicated the beneficial effects of NPs in *S. officinarum* plants during cold (Elsheery et al., 2020). nSi enhanced the photosynthetic pigments in *S. officinarum* plant leaves, which reveals that the NPs protected the plants from synthesizing different light-harvesting complexes, allowing the capture of large amounts of light energy, leading to enhanced photosynthetic responses (Ghafariyan et al., 2013; Verma et al., 2022a) (**Figure 1** and **Table 1**). nSi also enhanced the growth and development of *Solanum lycopersicum* plants during short-term cold stress (Elsheery et al., 2020).

Technological improvements can increase the production of agro-industrially, physiologically, and agronomically essential NPs that can in turn be utilized to produce fertilizers with decreased nutritional losses and improved nutrient use efficiency (NUE), employing smart delivery systems. NPs can be applied as nanofertilizers on the plants or in the rhizospheric soil to boost fertilizer uptake and utilization. They can also be used to upgrade plant development through advanced nanobiotechnology, supporting nutrient delivery systems with targeted approaches and multifunctional features for improved sustainable agriculture in years to come (Wang et al., 2022; Verma et al., 2022a). In addition, many of the aforementioned results have been obtained from field experiments, and thus, further exploration in hydroponics/soilless cultures is required. The study of how to adjust the levels and initial adaptive responses of physiological, biochemical, and molecular levels using nSi can be an interesting research field in the near future.

## Conclusions and future perspectives

Recent advanced sustainable agriculture strategies may be explored and applied to alleviate adverse environmental variables. Nowadays, the main objective of agricultural approaches is to promote plant production and quality. Hence, cost-effective technologies may be helpful for agri-farmers. Owing to their easy synthesis, maximum uptake, and large surface-to-volume ratio, nSi-based biofertilizers will be an excellent alternative to conventional synthetic fertilizers. Using nSi is an efficient approach for farmers to promote growth and production by increasing plant stress tolerance capacity. The drawback of using NPs on plants is DNA damage, which can occur through direct or indirect pathways. DNA damage occurs due to the degeneration of mitochondrial cristae, peroxisome proliferation, NO generation, and vacuolization. DNA repair processes are responsible for circumventing DNA damage; thus it also appears to be very important to evaluate the effects of NP exposure on these processes. The results presented above suggest that changes in stoichiometry are a potential morpho-functional adaptive response to NP exposure, caused by variations in the bioenergetic redox balance, which reduces the photosynthesis or cellular respiration rates.

Variations in nSi chemistry, size, shape, and electromagnetic properties lead to different findings on the effects and mechanisms of nSi in mitigating plant stresses. Earlier studies mainly discussed increasing physical barriers, growth promotion, inducing plant tolerance, and activating antioxidative enzymatic mechanisms, but rarely elucidated the impact of nSi on plant metabolites and the soil microbial community during stress conditions. Thus, the whole system should be considered, and in-depth omics research is needed on the nSi mechanisms that enhance a plant’s tolerance capacity to adversity, from the standpoint of physio-biochemical, transcriptomic, and proteomics levels. nSi-based fertilizers have opened up a whole new area of research opportunities for agro-scientists to synthesize advanced products that could help enhance agricultural productivity and reduce sustainable development challenges, without affecting the environment. The responsible application of nanotechnologies can hopefully play an important role in reaching this goal.

## Author contributions

KV and Y-RL conceptualized the article. All authors contributed to the article and approved the submitted version.
