# Erratum: Nanosilicon: An approach for abiotic stress mitigation and sustainable agriculture

**DOI:** 10.3389/fpls.2023.1177575

**Published:** 2023-03-22

**Authors:** Frontiers Production Office

**Affiliations:** Frontiers Media SA, Lausanne, Switzerland

**Keywords:** leaf gas exchange, enzymatic and non-enzymatic activities, abiotic stress, nano-silica, stress relief, environmental health

Due to a production error, the final copyedited version of this manuscript was not used.

The publisher apologizes for this mistake. The original version of this article has been updated.

